# High-Frequency Surface Insulation Strength with Nanoarchitectonics of Disiloxane Modified Polyimide Films †

**DOI:** 10.3390/polym14010146

**Published:** 2021-12-31

**Authors:** Zhaoliang Xing, Chong Zhang, Naifan Xue, Zhihui Li, Fei Li, Xiangnan Wan, Shaowei Guo, Jianhong Hao

**Affiliations:** 1 State Key Laboratory of Advanced Power Transmission Technology, Global Energy Interconnection Research Institute Co., Ltd., Beijing 102209, China; 15811444029@163.com (Z.X.); zhangc_sgri@163.com (C.Z.); lifei_geiri@163.com (F.L.); wanxiangnan@geiri.sgcc.com.cn (X.W.); m18826072964@163.com (S.G.); 2 State Key Laboratory of Alternate Electrical Power System with Renewable Energy Sources, North China Electric Power University, Beijing 102206, China; lzh_bryant@ncepu.edu.cn (Z.L.); jianhonghao@ncepu.edu.cn (J.H.)

**Keywords:** polyimide, disiloxane, high frequency creeping discharge, dielectric strength

## Abstract

High-frequency power transformers are conducive to the reliable grid connection of distributed energy sources. Polyimide is often used for the coating insulation of high-frequency power transformers. However, creeping discharge will cause insulation failure, therefore, it is necessary to use disiloxane for the purpose of modifying the molecular structure of polyimide. This paper not only introduces 1,3-bis(3-aminopropyl)-1,1,3,3-tetramethyldisiloxane (GAPD) with a molar content of 1%, 2%, and 5% to polyimide, but also tests both the physical and chemical properties of the modified film and the high frequency creeping dielectric strength. The results show that after adding GAPD, the overall functional groups of the material do not change, at the same time the transfer complexation of intermolecular charge and the absorption of ultraviolet light increase. There is no phase separation of the material and the structure is more regular and ordered, moreover the crystallinity increases. The overall dielectric constant and the dielectric loss tangent value show different trends, which means that the former value increases, while the latter value decreases. In addition, the resistivity of the surface and the volume increase, which is the same as the glass transition temperature. The mechanical properties are excellent, and the strength of bulk breakdown is mounting. The insulation strength of the high frequency creeping surface has been improved, which will increase with larger contents of GAPD. Among them, the relative change of the creeping flashover voltage is not obvious, and the creeping discharge life of G5 is 4.77 times that of G0. Further analysis shows that the silicon-oxygen chain links of the modified film forms a uniformly dispersed Si-O-Si network in the matrix through chemical bonds and charge transfer complexation. Once the outer matrix is destroyed, it will produce dispersed flocculent inorganic particles which have the role of protecting the inner material and improving the performance of the material. Combined with the ultraviolet light energy absorption, the increase of deep traps, the reduction of dielectric loss, and the improvement of thermodynamic performance, can better improve the high-frequency creeping insulation strength of polyimide film and its potential application value.

## 1. Introduction

Distributed energy has numerous advantages, such as small capacity, wide distribution, and cleanness, although it optimizes the power supply performance of the power grid, its instability brings challenges to system security [1]. Also known as power electronic transformers, the primary and secondary sides of high-frequency power transformers (HFPTs) are made up of highly controllable voltage amplitude and phase conversion circuits with power electronic devices, in this way the problems above can be effectively solved [2]. Polyimide (PI), as one of the best insulating organic materials to date, is commonly used in the coating insulation of HFPTs. The gas-solid insulation material inside HFPTs is in the high-frequency electrical stress environment affected by heat and electromagnetic coupling. Therefore, the insulation strength of polyimide is very important. The surface flashover field strength is much less than the air gap breakdown field strength of the same gap, which means that the insulation material is more likely to break down. Therefore, further improving the surface insulation strength of coated insulating materials is vital to ensure the development of HFPTs to a high voltage and large capacity.

The research shows that when modified by silicone, the polyimide has better properties such as high strength, high temperature resistance, high tensile strength, oxidation resistance, and a low expansion coefficient [3,4,5,6]. Moreover, the shorter the siloxane chain link is, the less likely it is to separate from the polyimide copolymer. Therefore, the shortest siloxane chain link-disiloxane should be used to modify the polyimide to theoretically obtain a polymer with no phase separation. It can be useful to optimize the modification effect and improve the stability of the material. At present, there is a lack of research on the molecular structure of disiloxane modified polyimide, especially on the test of the high-frequency surface insulation strength of the modified film and the analysis of the influence mechanism of modifications on its dielectric strength.

In this way, this paper introduces 1%, 2%, and 5% molar content of 1,3–bis (3-aminopropyl)-1,1,3,3-tetramethyldisiloxane into polyimide (GAPD) to modify its molecular structure. Firstly, its physical and chemical properties are characterized by infrared spectrum, UV-vis spectrum, scanning electron microscope, crystallinity, resistivity, dielectric properties, glass transition temperature, mechanical properties, and bulk breakdown strength. After this, the dielectric strength test including surface flashover voltage and creeping discharge life is carried out, and it describes the mechanism of disiloxane modification on its high-frequency surface dielectric strength, which provides a theoretical basis and optimization direction for the modification of high-frequency transformer coating insulation materials.

## 2. Film Preparation

The raw materials for the preparation of the modified film contain 4,4-diaminodiphenyl ether (ODA), pyromellitic anhydride (PMDA), N. N-Dimethylacetamide (DMAC), and 1,3-bis (3-aminopropyl)-1,1,3,3-tetramethyldisiloxane (GAPD), whose molecular formula are C12H12N2O, C10H2O6, C4H9NO, and C10H28N2OSi2, respectively.

**Figure 1:**
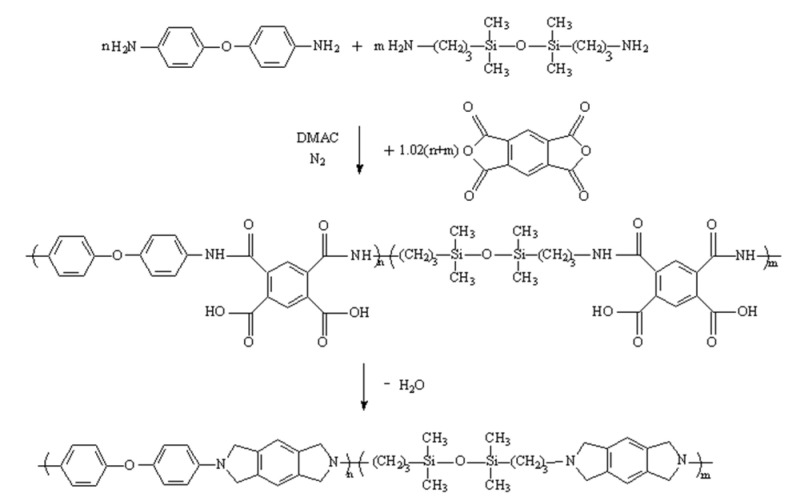
Polyimide reaction equation.

The films were synthesized using a two-step [7] method. Since the breakdown performance of composite dielectrics is generally optimal at low contents (<5wt.%) [8], based on the total molar content of ODA (15 mmol), GAPD with 1%, 2%, and 5% molar content were added to modify the molecular structure of polyimide. The pure polyimide is named G0, and the films are named G1, G2, and G5, respectively, according to their GAPD content. The polyimide reaction equation is shown in Figure 1. ODA and GAPD diamines were added and reacted with PMDA in DMAC to synthesize the polyamide acid solution (PAA). The PAA was further dehydrated and condensed to form the polyimide film.

**Figure 2:**
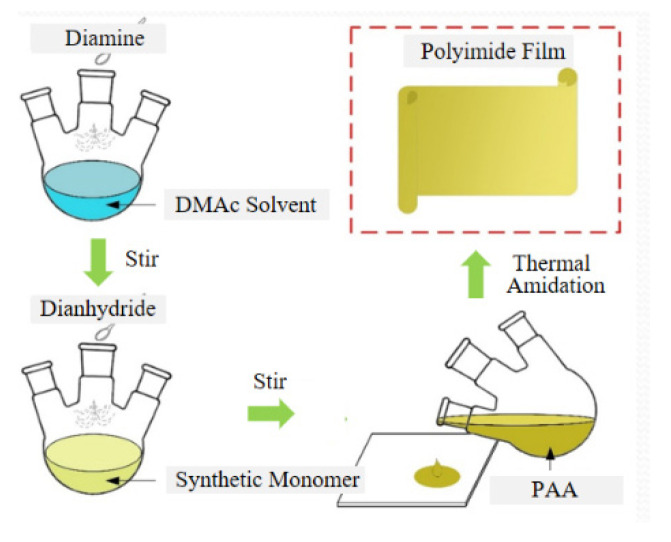
Process of film preparation.

The film preparation process is shown in Figure 2. First, under a nitrogen flow environment, a condenser tube was added to a three-port flask with a volume of 150 mL at the nitrogen outlet for solvent reflux. Then, it became 15 mmol diaminemonomer (ODA) after being filled with nitrogen, then GAPD monomers with different contents were added according to the sample modification scheme in Table 1. Next, this was followed by pouring in 35 mL of distilled and purified DMAC solution, putting it into an oil bath at 40 °C and stirring evenly, adding 1.02 times the molar content of diamine PMDA in the ratio of 6:3:1 every half an hour and stirring gently. After 12 h, a viscous golden PAA solution was obtained. Defoaming with mild circulating water for 0.5 h was conducted, then the glass sheet soaked in NaOH solution was taken out. Following this, the glass sheet was ultrasonically vibrated, rinsed, and dried with deionized water, then the surface was wiped with absolute ethanol and an applicator was used to evenly coat the glass with the viscous PAA solution. Then, it was vacuumed for 0.5 h, put into a high-temperature oven with natural air circulation for step heating (60 °C) × 2 h, 120 °C × 1 h, 200 °C × 1 h, 250 °C × 1 h, 320 °C × 0.5 h). After natural cooling, it was taken out and put in deionized water until the film was easy to remove. After drying, 28 ± 2 μM thick golden yellow polyimide film was produced.

The time from the appearance of corona on the film surface to the eventual occurrence of surface flashover is recorded as the material life. The statistical life of five films was obtained by using the Weibull statistical method, as shown in Table 1. It was found that after doping SiO_2_ particles, the lifetime increased with the increase of doping content. Among them, Si0 had the shortest lifetime and Si10 had the longest lifetime, which is 3.40 times that of pure polyimide. Therefore, in order to further study the effect of nano-composite modification of doped nanoparticles on the surface discharge characteristics of polyimide, the more representative Si0 and Si10 were photographed, and the surface discharge data were collected. 

## 3. Experimental Tests and Results

### 3.1. Infrared Spectrum

**Figure 3:**
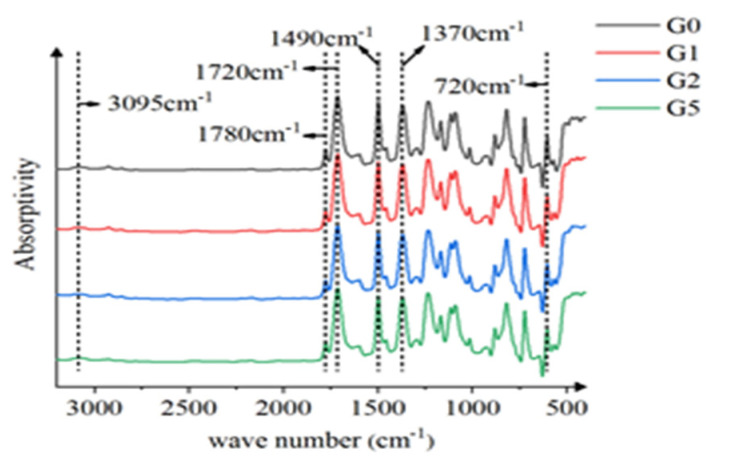
Infrared absorption spectrum.

In this paper, the infrared absorption spectra of the samples were measured by American Thermo Fisher Nicolet using the five Fourier transform infrared spectrometer, and different functional groups were determined to characterize their microstructure. The spectral range used is 4000–400 cm^−1^, and the infrared spectra of G0, G1, G2, and G5 are shown in Figure 3.

There are absorption peaks of the carbonyl group (C = O) in the imide ring at 1780 cm^−1^, 720 cm^−1^, and 1720 cm^−1^, and absorption peaks of the C-N-C group in the imide ring at 1370 cm^−1^. No absorption peaks appear near 1650 cm^−1^ and 1550 cm^−1^, indicating that all samples have completed thermal amidation after the heating process. There are characteristic absorption peaks of C-C bond and hydrocarbon C-H bond stretching in the benzene ring at 1490 cm^−1^ and 3095 cm^−1^, which means that the aromatic structure in the prepared films is intact. What must be pointed out is that the characteristic absorption peak of the silicon oxygen bond in all samples is not obvious due to less Si doping.

### 3.2. Ultraviolet Visible Spectrum

**Figure 4:**
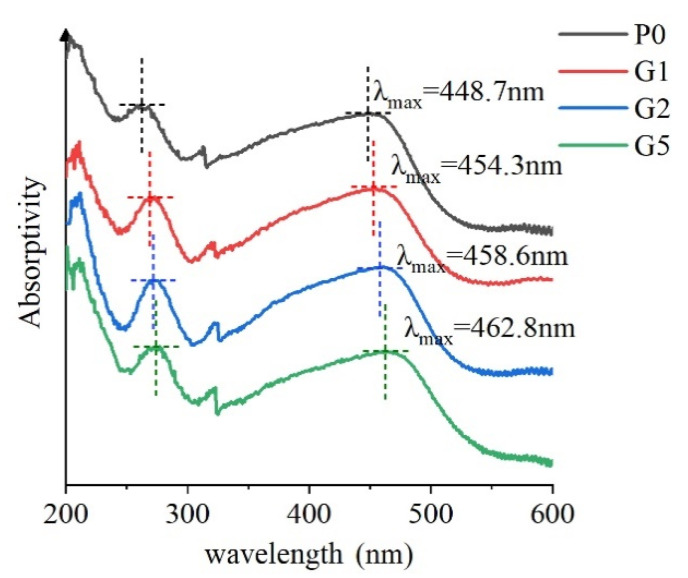
Ultraviolet visible absorption spectrum.

We used the Shimadzu (UV3600) ultraviolet visible spectrophotometer (UV-vis) to test the structural characteristics and charge transfer complexation (CTC) of the sample. The ultraviolet visible spectrum of 200~600 nm is shown in Figure 4.

Polyimide is composed of an electron donor (diamine link) and an electron acceptor (dianhydride link), charge transfer complexation between which will affect the stacking of polyimide molecules. Under the irradiation of ultraviolet visible light, electrons can make transitions between different orbitals. The wavelength corresponding to the position of maximum absorption on the spectral curve is defined as the maximum absorption wavelength λmax, which can be used to judge the moving direction of the spectral curve along the transverse axis. After the introduction of GAPD, the maximum absorption wavelength in the near ultraviolet region and visible region moves to the long wave direction, that is the red shift, which indicates that after the introduction of the GAPD link, the conjugation degree of the polymer system increases more, as a result of the stronger non bond electron transition between the electron donor and the electron acceptor of polyimide [9], that is the formation of a stronger charge transfer complex (CTC). Furthermore, the intermolecular force is increased, the regularity of the material is improved, and the stacking is more compact. In addition, the film has obvious color enhancement in the near ultraviolet region (200 nm–400 nm), showing that the silicon oxygen bond in the material absorbs more ultraviolet light. However, with a large angle and the higher energy and stability of the Si-O bond, the corrosion of ultraviolet light on the material matrix will effectively reduce.

### 3.3. Scanning Electron Microscope (SEM)

**Figure 5:**
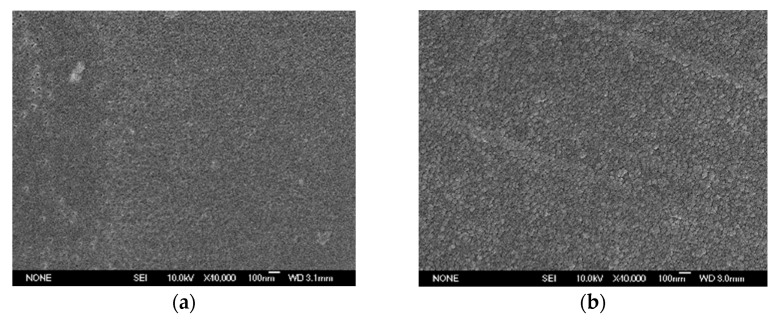
Surface morphology of the film. (**a**) G0, (**b**) G5.

**Figure 6:**
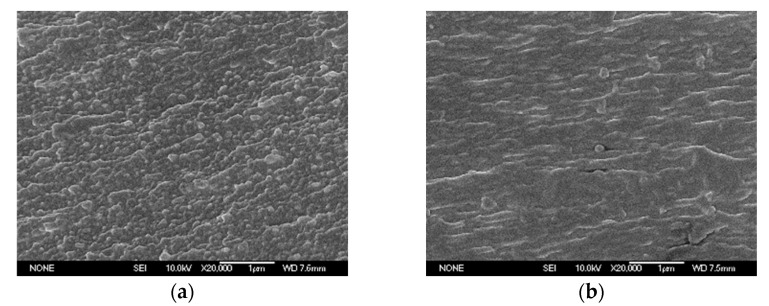
Cross-sectional morphology of the film. (**a**) G0, (**b**) G5.

The JSM-6700F scanning electron microscope (SEM) was used to observe the surface and section morphology of the sample. The surface of the film after gold spraying was enlarged by 40,000 times, as shown in Figure 5, and the section was enlarged by 20,000 times, as shown in Figure 6.

In terms of surface morphology, no phase separation was observed after the introduction of GAPD. In addition, after 40,000 times magnification, it was found that the matrix particles on the surface of G0 are tiny and there are many amorphous areas. However, with the increase of GAPD content, the matrix particle size increased significantly, the arrangement became more regular and compact, and the aggregation state of polyimide changed certainly. It can be seen from that the surface of the G5 matrix is relatively compact, and the aggregation state of the matrix is significantly different from G0. In terms of cross-section morphology, the mountain-like protrusion morphology caused by tensile fracture is easy to point out. After introducing GAPD, the cross-section morphology of G5 was similar to G0; the matrix is relatively uniform and no inorganic organic phase separation appears.

### 3.4. Crystallinity (DSC)

The crystallinity of the polyimide film was measured using the German Brooke D8 X-ray diffractometer (XRD). The test angle ranges from 5° to 90°, the scanning speed is 5°/min, and the analysis software is MDI jade 6.0. The polymer crystallinity is obtained by diffraction peak intensity/total intensity. Table 2 shows the crystallinity results obtained from the test.

Polyimide is a typical semi-crystalline polymer, which generally presents a state of short-range order and long-range disorder. In the solid state, there are both regular and irregular molecular arrangement regions, namely crystalline and amorphous regions. The percentage occupied is the crystallinity. Crystallinity is an important parameter to characterize semi-crystalline polymer materials, and it is directly related to many important properties of semi-crystalline polymers. Generally, the higher the crystallinity is, the more regular the molecular chains are arranged, and the more energy needed to break the molecular chains. As demonstrated in Table 2, in all the samples, the peak angles are in the range of 19° ~ 20°, and there is not an obvious law or trend of change. When GAPD is added, the overall crystallinity is higher than that of pure polyimide G0. Furthermore, GAPD is introduced the most in G5, so that the crystallinity is the highest. The reason for this phenomenon is shown through the results of ultraviolet-visible spectroscopy, after the introduction of GAPD, the intermolecular charge transfer complexation is enhanced, and the increase in the intermolecular force makes it easier for molecular chains to form regular and compact crystal regions with molecular arrangement, so the overall crystallinity is higher. The above results are also consistent with the results of scanning electron microscopy, that is, the introduction of GAPD will change the aggregation state of the polyimide matrix, and the structure is compact. 

### 3.5. Dielectric Properties

**Figure 7:**
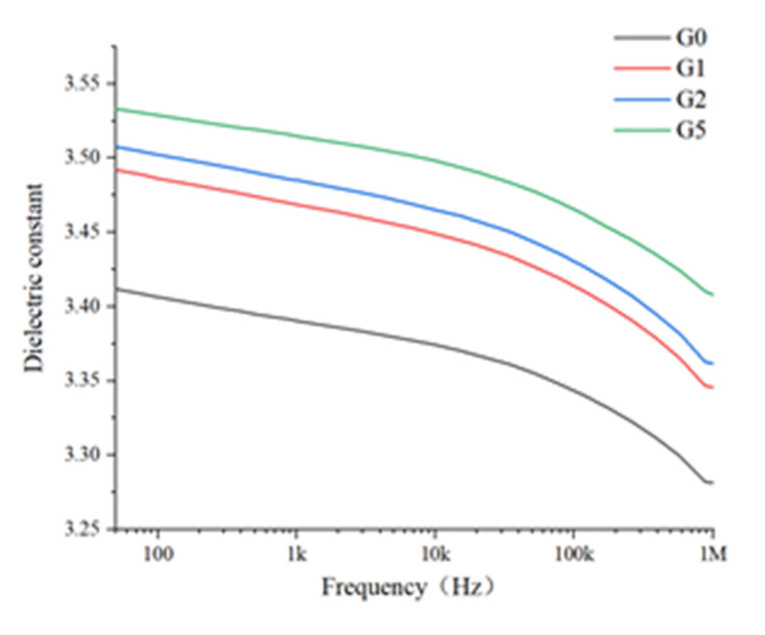
Relative permittivity of the films.

**Figure 8:**
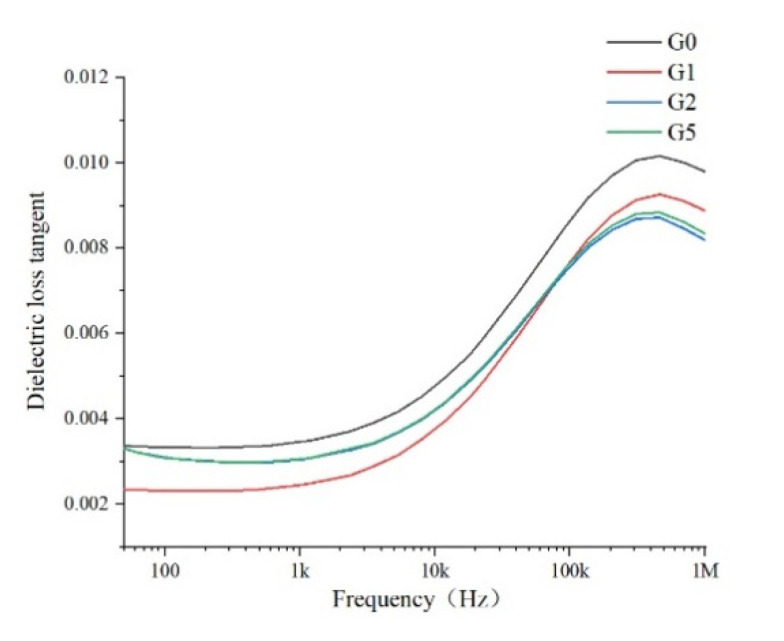
Dielectric loss tangent of the films.

The dielectric properties of the samples were tested using the Novocontrol dielectric constant tester. The dielectric response of the polyimide film after gold spraying was tested 10 times at 25 °C in the frequency range of 50 Hz–1 MHz. The average value of 10 tests of each sample was taken as the final test result, and the values of the dielectric constant as well as the dielectric loss tangent are shown in Figure 7 and Figure 8, respectively.

As a unique property of dielectrics, polarization is a physical phenomenon which means that the charge bound in the molecule cannot move completely freely in the dielectric and produce local migration under the action of an electric field to form induced dipole movement. It can be seen in Figure 7 that ranging from 50 Hz to 1 MHz, the relative dielectric constants of the four samples are in the range of 3.25~3.55, and all decrease with the increase in frequency. This is a common phenomenon and the reason for this is that when the frequency is low, the dipole in the material has sufficient polarization time, while in the high-frequency electric field, the polarization of the dipole cannot keep up with the change of the electric field, so the degree of polarization is low, which represents the decrease in the macro dielectric constant. For different samples, the relative dielectric constant of polyimide increased after adding GAPD, and the higher the content of GAPD added, the greater the increase is. This is because polyimide is a weakly polar polymer, the intermolecular force is strong, and the molecular steering is difficult to establish. However, research shows that the hindrance to the formation of the crystal lattice is small, the polarization is fully established, and the molecular polarization in the amorphous region is relatively difficult to establish. Therefore, after adding GAPD, the crystallinity of the sample increases, which leads to the high polarization ability of the modified material.

Except for conductivity loss, the dielectric also has periodic relaxation polarization related to thermal motion under an alternating electric field. As the conductivity of the polymer dielectric is quite low, the dielectric loss tangent is often used to characterize the dielectric loss characteristics of materials. It can be seen in Figure 8 that the dielectric loss characteristics of the four samples are slightly different in the low frequency region and greatly different in the high frequency region. With the increase of frequency, the tangent value increases continuously in the range of 50 Hz to 1 MHz and decreases slightly in the later stage, and the tangent of the frequency dielectric loss increases slowly in the low frequency range. This is because the dipole can keep up with the change of electric field in the low frequency region, so the dielectric loss is low. With the increase in frequency, the polarization cannot keep up with the change of electric field in the high frequency region, and the dipole steering is affected by resistance, leading to the increase in dielectric loss [10]. When the frequency is much too high, the relaxation polarization is too late to be established, and the displacement polarization is the dominant one, thus the maximum value appears. For the four samples, in the high-frequency range (1 kHz ~ 100 kHz), the addition of GAPD leads to an obvious reduction of dielectric loss, this is because the addition of GAPD causes an increase in polymer crystallinity, which is conducive to the polarization steering of dipole to reduce the heat generation caused by resistance under an alternating electric field.

### 3.6. Resistivity

**Figure 9:**
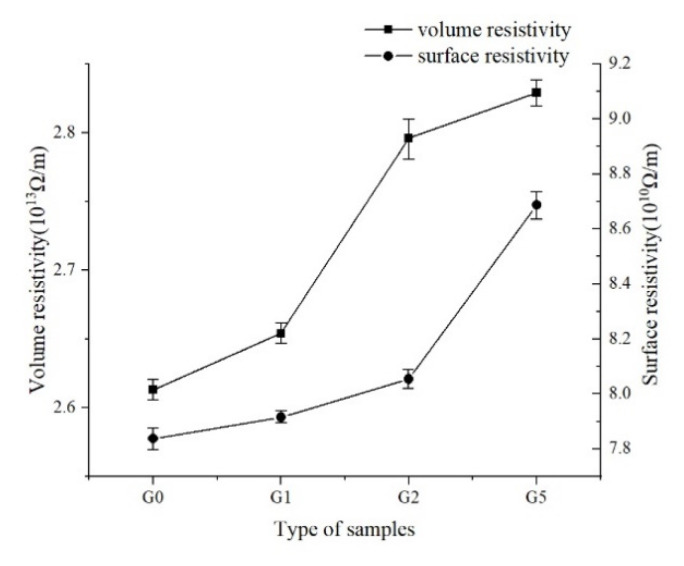
Surface resistivity and volume resistivity.

According to the standard IEC60093-1980<Methods of test for volume resistivity and surface resistivity of solid electrical insulating materials> [11], the volume and surface resistivity of the polyimide film were tested with the American Keithley 6517B megger and the 8009 three electrode fixture. Before the test, the insulating material should be electrically aged for 1 min and the indication should be read after it is stable. The test results are shown in Figure 9.

The figure shows that the addition of GAPD leads to an increase in surface and volume resistivity. With the increase of the addition content, the resistivity of polyimide increases more obviously. Polyimide itself is composed of an amorphous phase and a crystalline phase, and its conductance is mainly composed of electronic hopping conductance. Therefore, after adding GAPD, the crystallinity increases, the aggregation state changes, and the distance between the microcrystalline phase decreases. Moreover, the phase barrier increases, which increases the volume resistivity. Surface resistivity is not only related to the nature of the medium itself, but also greatly to the external environment (such as humidity, contamination, and so on). Silicone makes the material hydrophobic and hinders the influence of water in the air on the electron conduction at the gas-solid interface, so the surface resistivity of polyimide is significantly improved.

At the same time, polyimide is a weak polar dielectric, and the loss includes a part of the loss caused by the impurity conductivity. This part of the loss can be referred to as the gas dielectric, and the dielectric loss is proportional to the volume conductivity. The addition of GAPD leads to the decrease in conductivity, which also further reduces the dielectric loss of polyimide after the addition of GAPD.

### 3.7. Differential Scanning Calorimeter (DSC)

**Figure 10:**
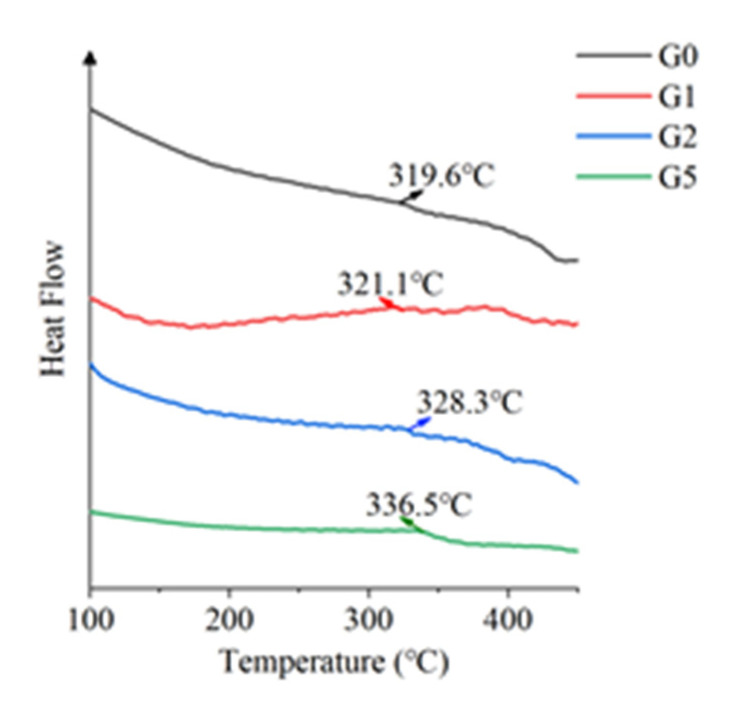
Secondary heating curve.

The glass transition temperature (Tg) of the sample was measured using the German Niche 200F3 differential scanning calorimeter (DSC), to characterize its thermal properties. The extrapolated initial temperature is taken as Tg, and the secondary heating curve after the initial thermal cycle is shown in Figure 10.

Tg is not only the lowest temperature, but also the upper limit of working temperature for the free movement of amorphous polymer macromolecular segments. The increase of Tg after adding GAPD is due to the fact that the addition of GAPD will affect the free movement of polymer macromolecules, strengthening the charge transfer complexation, and enhancing the intermolecular force, which makes it more difficult for the polyimide molecular chain to convert into a state of free movement.

### 3.8. Mechanical Strength Testing

**Figure 11:**
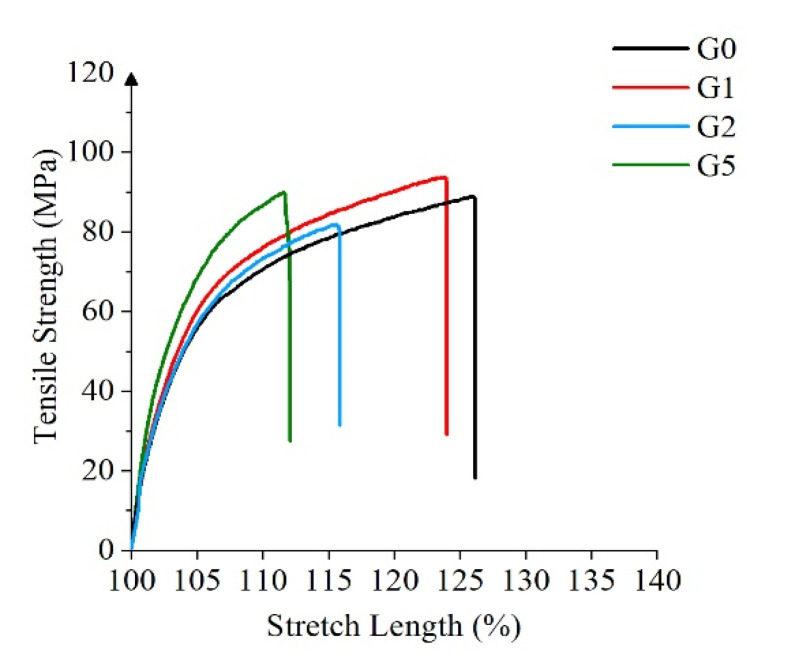
Mechanical strength.

The mechanical properties of the samples were tested using the high-speed rail testing instrument A1-7000M tensile testing machine. Five groups of tests were carried out on each film. Finally, the median was taken as the test result. Figure 11 shows the curves of the tensile strength of the four samples with the stretch length.

The addition of GAPD contributes to the decrease of the maximum stretch length, but the maximum tensile strength grows. This is because the addition of GAPD changes the aggregation state of polyimide, which has the characteristics of a compact structure, more orderly molecular arrangement, and is rigid and brittle. The overall mechanical properties are excellent. The maximum tensile strength is maintained above 80 MPa, and the minimum elongation at break is still above 11%, which meets the engineering application requirements.

### 3.9. Bulk Breakdown Strength Test

**Figure 12:**
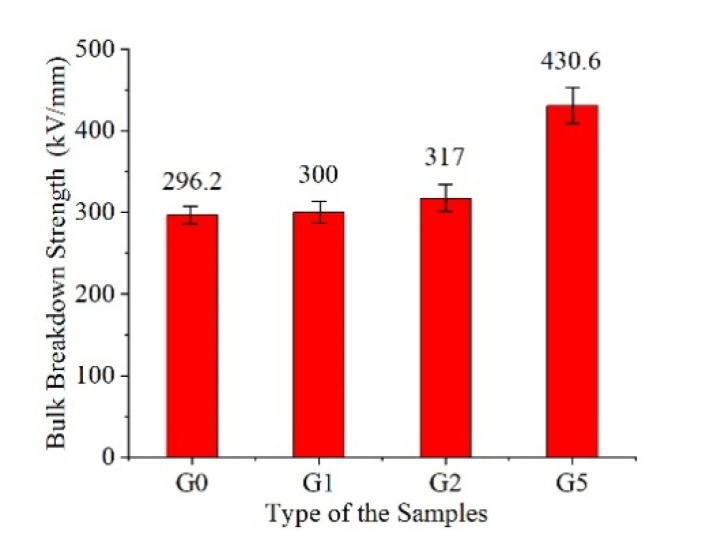
Bulk breakdown strength.

The film was cut into a 40 mm × 40 mm square and placed between the equal diameter cylindrical electrodes in silicone oil. The film was pressurized at 1 kV/s until broken down. The results are shown in Figure 12.

As shown in the above figure, the bulk breakdown strength of medium-purity polyimide is 296.15 kV/mm, which is similar to that of Kapton product manual (303 kV/mm). The addition of GAPD has benefits for improving the DC bulk breakdown strength of the film. Furthermore, the content and the bulk breakdown strength are positively correlated. Finally, the breakdown strength of G5 is the largest, which is 1.45 times that of G0.

### 3.10. High-Frequency Creeping Discharge Experiment

**Figure 13:**
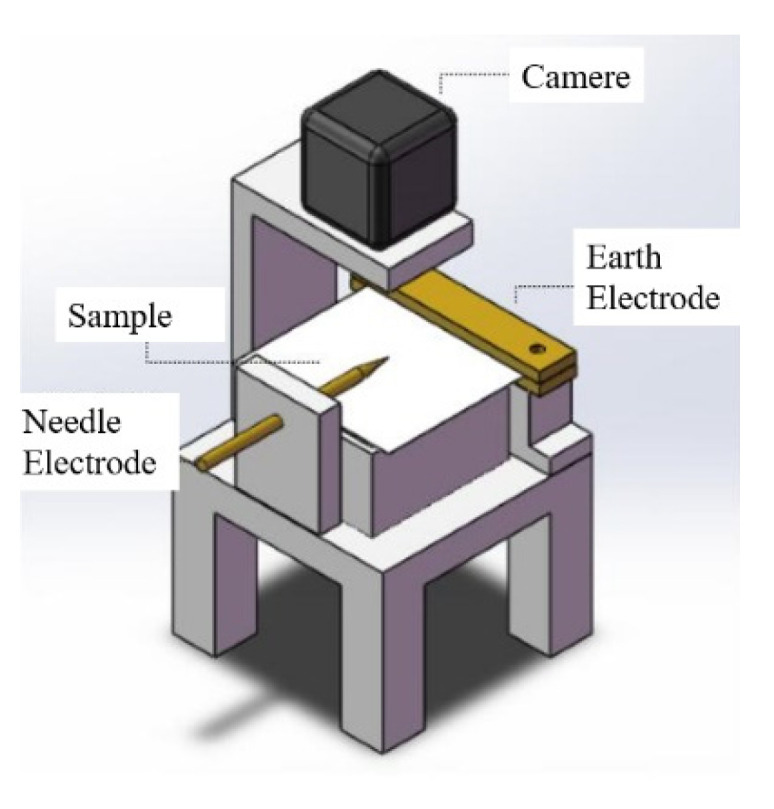
High-frequency surface discharge experimental system.

The high-frequency creeping discharge experiment of the sample was carried out using a needle-plate electrode. The experimental system is shown in Figure 13. The sample film with a size of 5 cm × 5 cm was placed flatly under the needle electrode and on the Kapton polyimide pad. The experimental temperature was 20 °C and the relative humidity was 25%. The experimental voltage frequency chosen was 20 kHz.

**Figure 14:**
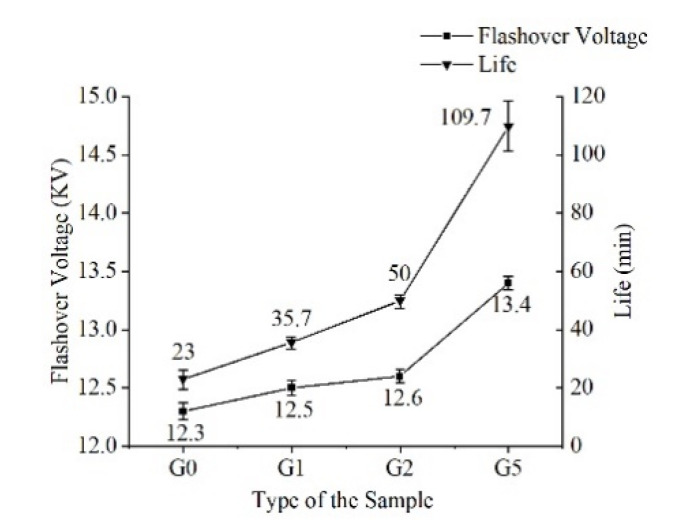
High-frequency surface insulation strength.

The flashover voltage was measured, and the sample was pressurized at the rate of 0.2 kV/s until surface flashover, which was recorded as flashover voltage. The surface discharge life was measured under the condition of applying the high-frequency sinusoidal voltages of 11 kV and 20 kHz. The time from pressurization to film breakdown was recorded; this is defined as the creeping discharge life. The flashover voltage and lifetime of the samples were characterized by Weibull distribution, as shown in Figure 14.

The Figure 14 shows that the flashover voltage increases slightly after introducing GAPD, yet the amplitude is not obvious. This is because the flashover voltage at the gas-solid interface is unstable and the process is rapid, which is greatly affected by air. In addition, the high-frequency creeping discharge life of polyimide increases, and the higher the content added, the longer the life becomes. The Weibull statistical life of G5 is 4.77 times that of G0. Overall, the high-frequency creeping insulation strength of G5 is the highest.

**Figure 15:**
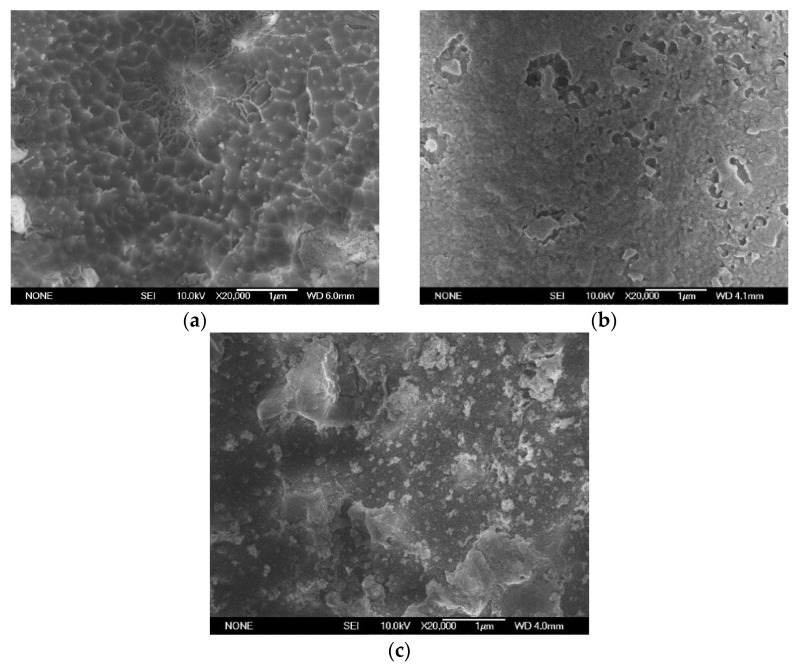
Surface morphology of materials after discharge. (**a**) G0-10 min, (**b**) G5-10 min, (**c**) G5-40 min.

To conduct a further study on the influence mechanism of the high-frequency creeping insulation strength of materials, 11 kV and 20 kHz high-frequency sinusoidal voltages were used to electrically age G0 and G5 for 10 min, and G5 for 40 min, with the changes in material surface morphology being observed by SEM, as shown in Figure 15.

After adding high-frequency voltage for 10 min, the surface layer of G0 was seriously damaged, local pore groups were formed, the polyimide skeleton was exposed, and the pore groups had the tendency of connecting. After G5 was pressurized for 10 min, most of the surface layer remained flat, and the larger matrix particles stacked together smoothly. After G5 was pressurized for 40 min, the surface layer was destroyed in a large area, the organic molecules were destroyed and disappeared, and the remaining flocculent particles were evenly dispersed on the surface layer, simultaneously exposing the relatively flat inner layer matrix. 

## 4. Analysis of Modification Mechanism

After adding GAPD, the physical properties, chemical properties, and high-frequency surface insulation strength of polyimide films changed, and the influence mechanism for the modification of materials needs further analysis.

**Figure 16:**
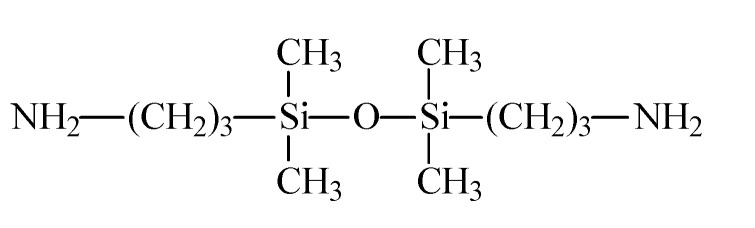
Molecular structure of GAPD.

**Figure 17:**
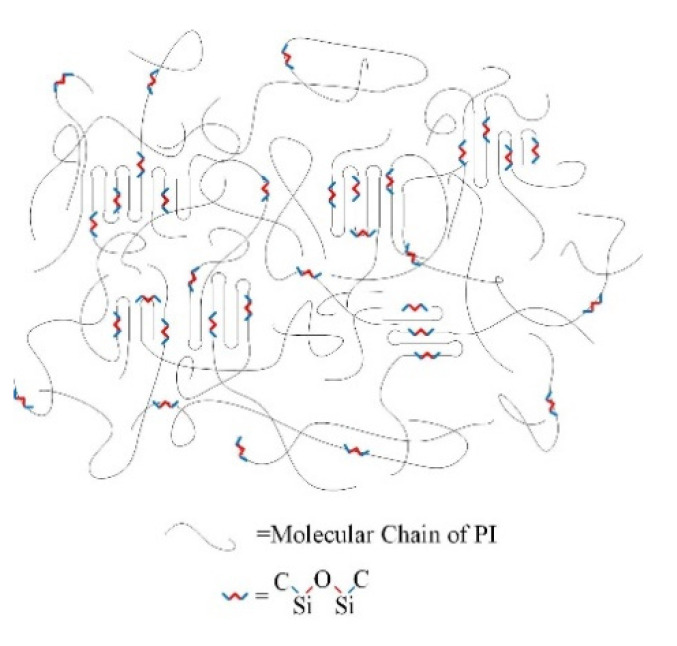
Structural model of modified materials.

GAPD is a diamine containing Si-O-Si chain, whose molecular structure is shown in Figure 16. The addition of GAPD forms polyimides which are different from the traditional stacking structure, causing more complex changes in material properties. The structural model of the material is shown in Figure 17. The Si-O-Si chain is evenly distributed between the aggregated state and the non-aggregated state, and the Si-C bond at both ends is bonded with polyimide. The Si-O-Si network is cross-linked into a more uniform and stable Si-O-Si network through the intermolecular charge transfer complexation and Van der Waals forces, which effectively improves the compatibility of the Si-O bond with the polyimide matrix, increases the density of the network, and optimizes the physical and chemical properties of the polymer. Except for this, the dispersed Si-O bond in the matrix has high bond energy and high strength, which also enhances the insulating properties of the polyimide molecular chain itself.

It can be seen from the analysis of the physical and chemical properties of the above materials that under certain conditions the main functional groups do not change, the inorganic-organic phase do not separate, the matrix aggregation state after access to GAPD changes, the material structure becomes more compact, the UV absorption of the Si-O structure expands, the resistivity and crystallinity increase, the tangent angle of mechanism loss decreases, and the Tg value and maximum tensile strength climb. The electrons produced by corona discharge, ultraviolet light, and local high temperature cause insulation breakdown of the material. Therefore, the influence mechanism of the physical and chemical properties of the semi-crystalline polymer matrix on the surface insulation strength, including instantaneous and long-term high-frequency, is divided into four aspects. At the beginning, the study showed that the electrical properties of the semi-crystalline polymer strongly depend on its aggregated structure, the charge transfer complexation is on the rise, and the material structure is more uniform and compact, which will effectively hinder the carrier movement and delay the formation of conductive channels [12] so that it is more difficult to form electronic collapse. Moreover, the dense structure is not conducive to the free movement of the molecular chain after capturing the charge, and it is difficult to accumulate energy to destroy the material. Secondly, with short UV wavelength and high energy, the absorption of UV light by Si-O structure expands, which will reduce the absorption of energy by the material matrix, effectively reduce the photoionization degree on the material surface, and inhibit the erosion of UV light on the matrix. Additionally, polyimide is a typical semi-crystalline polymer. According to the “two-phase structure” model, the intermolecular force in the crystalline region is strong, and the amorphous region is located between the crystalline regions. The trap distribution of the film is closely related to its local state, research shows [13] that the crystalline phase will lead to a relatively deep trap, and due to the strong intermolecular force, the electrons will interact with the surrounding molecular chains, resulting in the local polarization effect and the “self-trapping” phenomenon [14], further increasing the depth of the trap. The amorphous region will create shallow traps, and the number of shallow traps is relatively large due to the high activity of the amorphous region. The trapping and collapsing of charge have great influence on the discharge behavior of the material surface. According to the bipolar carrier model [15], the chemical defects introduced by molecular structure of modified polyimide are closer to the Fermi level. The deep trap barrier is large, and the charge is difficult to collapse after trapping, so it is easy to form stable space charge. The trapped charge has the same polarity as the applied electric stress, which weakens the original electric field and reduces the distortion of the electric field. In addition, thermal aging will cause the fracture of the chemical bond of polyimide. On the one hand, the tangent value of the dielectric loss angle of polyimide decreases significantly after the addition of GAPD, resulting in less heat generated by the modified material under the same conditions. On the other hand, the increase in the Tg value of the modified material indicates that the material is more difficult to change from glass state to high elastic state, and the structure of the material itself is more difficult to change by external stress. Both of the aspects will reduce the destructive effect of heat on the material. 

From the observations on surface morphology after discharge of G5 in Figure 15, it can be found that the surface layer of G5 is less damaged after being subjected to high-frequency creeping discharge shock in the same length of time. Even if the surface is destroyed, the exposed next layer is still tight and smooth. Even though most of the outer layer is destroyed, agglomerated flocculent particles remain on the surface of the inner matrix. According to the probability formula of initial electron generation in Richardson-Schottky’s law [16], it can be seen that in the high-frequency creeping discharge, the corona continues to erode the surface of the material, and the creeping discharge will be more intense with the deepening of the surface damage of the material, so that the active material is exposed and reacts with the oxygen atoms ionized in the air, which makes the open-loop reaction of the polyimide molecular chain occur. Compared with the Si-C bond and C-C bond, the energy of the Si-O bond is the largest, so it is finally broken and forms a phase-separated inorganic silicon oxide in the air. Furthermore, the siloxane oxidation crosslinking reaction occurs in polyimide containing a siloxane structure at high temperature generated by creeping discharge, and the structural phase similar to silica appears on the surface [17]. The material is finally transformed from an organic structure into an inorganic/organic phase separation structure. The inorganic structure can also effectively scatter electrons in order to protect the inner material, which improves the mechanical strength, heat resistance, and insulation strength of the inner material.

## 5. Conclusions

In this paper, the molecular structure of polyimide is modified by introducing 1%, 2%, and 5% molar content of disiloxane (GAPD). The physical and chemical properties and high frequency creeping insulation strength of the modified film are tested. The following conclusions were generated:

(1) After the addition of GAPD, the overall functional group structure of polyimide remained unchanged, and the intermolecular charge transfer complexation was enhanced. The absorption of UV energy increased. The aggregation state of the polyimide matrix changed, and the crystallinity increased. The matrix structure became more compact and there was no phase separation. The relative dielectric constant increased, while the tangent value of the dielectric loss angle dropped. In addition, the surface resistivity and volume resistivity grew. Moreover, the glass transition temperature rose, the mechanical properties were excellent, and the bulk breakdown strength increased.
(2) After the addition of GAPD, the high-frequency creeping insulation strength of the film improved and had the same tendency as the content. The increase in creeping flashover voltage was relatively insignificant, and the life of creeping discharge was larger. G5 had the longest statistical life, which is 4.77 times that of G0.
(3) The influence mechanism of the modification of polyimide modified by disiloxane molecular structure on the high-frequency creeping insulation strength of the film was preliminarily clarified. The molecular structure modification formed a uniformly distributed and non-phase separation Si-O-Si network through chemical bonds, charge transfer complexation, etc. After the destruction of the outer substrate, the dispersed flocculent inorganic particles were produced to protect the inner material and improve the performance of the material. The multi-factor synergy can more effectively improve the high-frequency creeping insulation strength of the material.

## Figures and Tables

**Table 1:** Modification scheme.

Film Type	Monomer Content		
	GAPD (%)	ODA (mmol)	PMDA (mmol)
G0	0	15	15.3
G1	1	15	15.453
G2	2	15	15.606
G5	5	15	16.065

**Table 2:** Quantitative statistics of creeping discharge.

Film Type	G0	G1	G2	G5
Crystallinity (%)	12.79	12.19	12.86	14.93
Peak angle (°)	19.83	19.86	19.58	19.13
